# *EGR1* and *KLF4* as Diagnostic Markers for Abdominal Aortic Aneurysm and Associated With Immune Infiltration

**DOI:** 10.3389/fcvm.2022.781207

**Published:** 2022-02-09

**Authors:** Chunguang Guo, Zaoqu Liu, Yin Yu, Zhibin Zhou, Ke Ma, Linfeng Zhang, Qin Dang, Long Liu, Libo Wang, Shuai Zhang, Zhaohui Hua, Xinwei Han, Zhen Li

**Affiliations:** ^1^Department of Endovascular Surgery, The First Affiliated Hospital of Zhengzhou University, Zhengzhou, China; ^2^Department of Interventional Radiology, The First Affiliated Hospital of Zhengzhou University, Zhengzhou, China; ^3^Department of Pathophysiology, School of Basic Medical Sciences, The Academy of Medical Science, Zhengzhou University, Zhengzhou, China; ^4^Department of Colorectal Surgery, The First Affiliated Hospital of Zhengzhou University, Zhengzhou, China; ^5^Department of Hepatobiliary and Pancreatic Surgery, The First Affiliated Hospital of Zhengzhou University, Zhengzhou, China

**Keywords:** perivascular adipose tissue, abdominal aortic aneurysm, diagnostic marker, immune infiltration, machine learning

## Abstract

**Background:**

Formation and rupture of abdominal aortic aneurysm (AAA) is fatal, and the pathological processes and molecular mechanisms underlying its formation and development are unclear. Perivascular adipose tissue (PVAT) has attracted extensive attention as a newly defined secretory organ, and we aim to explore the potential association between PVAT and AAA.

**Methods:**

We analyzed gene expression and clinical data of 30 PVAT around AAA and 30 PVAT around normal abdominal aorta (NAA). The diagnostic markers and immune cell infiltration of PVAT were further investigated by WGCNA, CIBERSORT, PPI, and multiple machine learning algorisms (including LASSO, RF, and SVM). Subsequently, eight-week-old C57BL/6 male mice (*n* = 10) were used to construct AAA models, and aorta samples were collected for molecular validation. Meanwhile, fifty-five peripheral venous blood samples from patients (AAA vs. normal: 40:15) in our hospital were used as an inhouse cohort to validate the diagnostic markers by qRT-PCR. The diagnostic efficacy of biomarkers was assessed by receiver operating characteristic (ROC) curve, area under the ROC (AUC), and concordance index (C-index).

**Results:**

A total of 75 genes in the Grey60 module were identified by WGCNA. To select the genes most associated with PVAT in the grey60 module, three algorithms (including LASSO, RF, and SVM) and PPI were applied. *EGR1* and *KLF4* were identified as diagnostic markers of PVAT, with high accurate AUCs of 0.916, 0.926, and 0.948 (combined two markers). Additionally, the two biomarkers also displayed accurate diagnostic efficacy in the mice and inhouse cohorts, with AUCs and C-indexes all >0.8. Compared with the NAA group, PVAT around AAA was more abundant in multiple immune cell infiltration. Ultimately, the immune-related analysis revealed that *EGR1* and *KLF4* were associated with mast cells, T cells, and plasma cells.

**Conclusion:**

*EGR1* and *KLF4* were diagnostic markers of PVAT around AAA and associated with multiple immune cells.

## Introduction

Abdominal aortic aneurysm (AAA) is a pathological and progressive dilatation of the infrarenal abdominal aorta, which results in an increasing risk of rupture. Ruptured AAA is lethal and usually asymptomatic until they rupture, with a mortality of 85–90% (1). AAA is a multifactorial disease, caused by the combination of genetic, biochemical, and environmental factors. Smoking, male sex, age older than 65 years, obesity, and positive family history are the major risk factors of AAA (2, 3). During the AAA formation and progression, the three-layered vascular wall of the abdominal aorta gradually expands and weakens (4). Inflammatory cell infiltration, oxidative stress, vascular smooth muscle cell (VSMC) depletion, and destructive extracellular matrix are the primary pathophysiology mechanism of AAA (5, 6).

Although more than 200,000 people die of AAA rupture every year and the number was gradually increasing, the treatment approach of AAA is relatively scarce (7). Medical treatment is ineffective for AAA, and patients only have the option to receive surgery or endovascular repair before the aneurysm ruptures, although this has a relatively high operation risk and the possibility of postoperative complications, such as hemorrhagic shock, stent leakage, and infection (8–10). Consequently, the development of effective treatment modalities to delay and prevent the expansion or rupture of AAA is an urgent issue. To complete this task, further research to understand the deeper mechanisms is needed.

Perivascular adipose tissue (PVAT) is a unique tissue in organism and initially thought to provide mechanical support for the vessel wall, which surrounds most blood vessels including the abdominal aorta (5, 11). Multicenter research showed differences in PVAT distribution for patients with AAA. Compared with the NAA, excessive accumulation of PVAT in the aorta vessel wall is an independent risk factor for AAA formation and rupture (12). It is well established that obesity will promote the development of AAA and vascular occlusive disease, due to its role as a risk factor for atherothrombosis (13). Noteworthy, compared with visceral fat in other areas (subcutaneous abdominal and omental visceral fat), PVAT is particularly significant in the enrichment of inflammatory factors, which possibility is potential link with the expansion and weaken of the abdominal aorta wall (11, 14, 15). Additionally, aorta PVAT was unequivocally associated with abdominal aorta diameter in individuals. This correlation persisted after adjustment for gender, age, positive family history, and obesity (13, 14). PVAT is a fat depot with highly active endocrine and paracrine functions that is possibly responsible for the phenomenon. In particular, adiponectin and *H*^2^*S* released by PVAT have been shown to advance the inflammatory response and promote vasodilation. Therefore, the importance of PVAT in the formation to rupture of AAA is unquestionable and deserves further attention (16).

A more recent study has demonstrated that the pathogenesis of AAA was associated with immune cells from PVAT (5). There was immune cell infiltration in the lesion of AAA. T cells are the most important component, and B cells, macrophages, and NK cells also account for a proportion (17). Noteworthy, with the diameter of AAA increased, the degree of immune cell infiltration in the PVAT and abdominal aorta wall was concomitant elevated (5, 18). In addition, artery tertiary lymphoid organs (ATLOs) and pericardial adipose tissue have a regulatory effect on the accumulation of immune cells (19, 20). For the aforementioned reasons, we hypothesize that PVAT serves as an adipose-associated ATLO plays an important role in the cardiovascular pathophysiology. Herein, to elaborate the molecular mechanism of PVAT and develop new immunotherapeutic targets, it is indispensable to assess the infiltration of immune cells and analyze the differences in immune cell composition between PVAT around AAA and NAA. In recent, a versatile computational method, termed CIBERSORT, was developed to quantify the relative levels of 22 immune cell types in the tissues. CIBERSORT has been widely used in cardiovascular and cerebrovascular diseases (including stroke and atherosclerosis) (21, 22), but no study has used it in PVAT.

In this study, the analysis of the differentially expressed genes between PVAT around AAA and NAA was performed and then identified diagnostic markers of PVAT by multiple machine learning algorithms. *EGR1* and *KLF4* were identified as biomarkers and validated in mice and inhouse cohorts. ROC curve and C-index were used to assess the diagnostic efficacy of the two genes. Subsequently, immune-related pathways were enrichment, and abundance of 22 immune cells was compared by gene set enrichment analysis (GSEA) and CIBERSORT. Ultimately, we focused on the relationship between infiltrating immune cells and diagnostic markers to reveal the immune molecular mechanism of PVAT more comprehensively around AAA.

## Materials and Methods

### Data Source and Preprocessing

**Figure 1:**
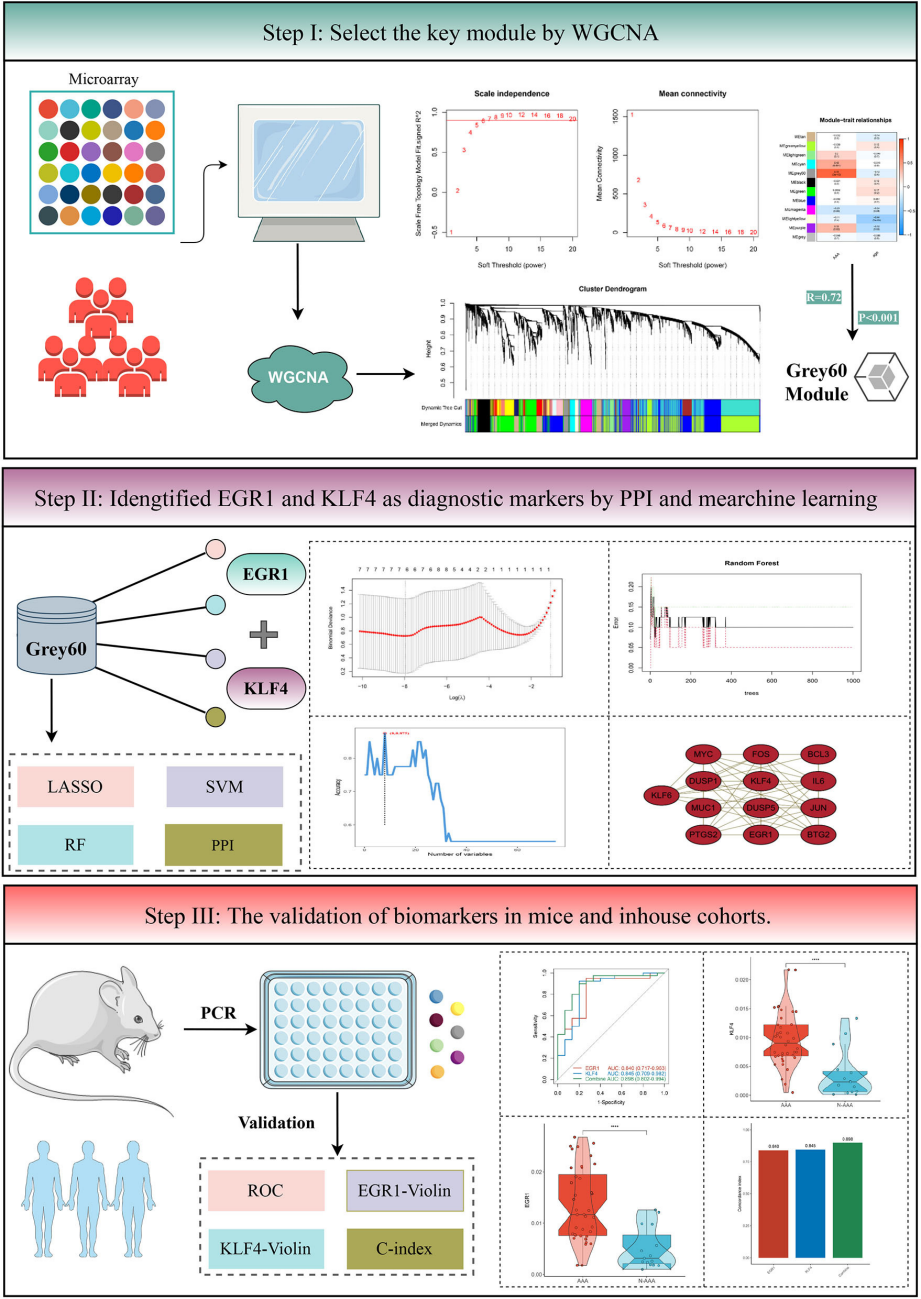
The flowchart of analysis procedure.

The details of overall analysis flowchart are shown in Figure 1. In the beginning, clinical annotation and gene expression profiling by array data of GSE119717 (14) were retrieved from Gene Expression Omnibus (GEO) database. This dataset belonged to Illumina^®^ platforms (GPL10558), including PVAT around the AAA or NAA (30 vs. 30 samples). The raw data were processed and normalized using the “limma” (version 3.46.0) (23) R package. Gene annotations for each probe set were derived from the Ensembl database. If multiple probe sets represent the same gene, the probe set with the highest mean intensity across all samples was retained.

### Weighted Gene Coexpression Network Analysis

Weighted gene coexpression network analysis (WGCNA) was applied to summarize gene expression data into coexpression modules. WGCNA has an obvious advantage to reveal the complex relationships among modules, networks, and phenotypes. In detail, based on gene expression profiles and GPL annotation data, a total of 14,016 genes were identified from each sample in this study. The top 5,000 genes were selected according to the median absolute deviation (MAD) of each gene. Then, the top 5,000 genes were used to construct a coexpression network by “WGCNA” (version 1.70–3) R package (24). To reach the goal of scale-free network distribution, the Pearson's correlation coefficient cor (i, j) was used to acquire the similarity matrix by calculating the link strength between nodes i and j from coexpression network and selecting the optimum soft threshold β. Next, the weighted adjacency matrix was constructed as follows: a_ij_ = [0.5 × (1 + cor (i, j))]^β^. Subsequently, the adjacency matrix was transformed into topological overlap matrix (TOM), representing the overlap of network neighbors, and (1-TOM) described the dissimilarity between genes to identify hierarchical clustering nodes and modules. Eventually, the hierarchical clustering results were partitioned by dynamic tree algorithm (deep-split = 2; merge module height = 0.30; minimum module size = 30; cut tree height = 0.99). To evaluate and investigate the coexpression similarities of all modules, the dissimilarity of module eigengene was calculated. Finally, modules with a correlation of 0.70 were merged by “mergeCloseModules” function with essential parameters.

### Clinically Significant Modules

The MEs were used for the component analysis of each module, and modules with similar expression profiles showed highly correlated eigengenes. The correlation between coexpression modules and clinical traits was estimated based on the phenotypic information of adipose tissues around the abdominal aorta. The gene module with the highest correlation coefficient and *p* < 0.05 was considered the most relevant module to ischemic events and was defined as the key module.

### Functional Enrichment Analysis

Functional enrichment was assessed using Gene Ontology (GO) and Kyoto Encyclopedia of Genes and Genomes (KEGG) pathway enrichment analysis. The process was complete by “clusterProfiler” (version 3.18.1) R package (25). The terms with false discovery rate (FDR) < 0.05 were considered significant.

### Protein–Protein Interaction Network Construction

Protein–protein interaction (PPI) is a protein network analytic algorithm, which was used to better understand molecular function and cellular mechanisms, whereas network analysis is an effective method to reveal all kinds of PPI networks. Here, we submitted all genes within the key module to the STRING (version 11.0; https://string-db.org/) database with a minimum level of confidence >0.4 (26, 27). Then, PPI networks were constructed by Cytoscape software (version 3.8.0; http://www.cytoscape.org) (28, 29). Based on maximal clique centrality algorithm, significant models and hub genes were calculated and selected by molecular complex detection (MCODE) which was a plugin for Cytoscape; parameters setting for MCODE: maximum depth = 100, node score cut ≥2, K-core ≥2, degree cut ≥2.

### Screening and Verification of Diagnostic Markers

We used random forest (RF), least absolute shrinkage and selection operator (LASSO) logistic regression, and support vector machine-recursive feature elimination (SVM-RFE) to identify key biomarkers for PVAT. RF is a nonparametric, supervised method of classification (30). It is a combination of tree predictors and with the same distribution for all trees in the forests. The RF algorithm was applied with the “RandomForestSRC” package. LASSO regression has a contractile penalty function on variables that induces sparsity of predictors in the expression profile. To achieve this purpose, the “glmnet” package was applied (31). Furthermore, SVM-RFE is used to find the best variables by deleting SVM-generated eigenvectors, which is a machine learning method based on support vector machine. To further identify the diagnostic value of biomarkers in PVAT, SVM module was developed by the “e1071” package (32). Ultimately, we combined the genes from the three machine learning algorithms for further analysis. Bilateral *p* < 0.05 were considered as statistically significant. The diagnostic efficacy of biomarkers was assessed by receiver operating characteristic (ROC) curve, area under the ROC (AUC), and concordance index (C-index).

### Development of the AAA Mice Model

This project was approved by the Ethics Committee Board of The First Affiliated Hospital of Zhengzhou University. According to a previous study (33), the eight-week-old C57BL/6 male mice (*n* = 10) were fed with normal diet. A total of 0.2% 3-aminopropionitrile fumarate salt drinking water was provided to mice 2 days before surgery until the end of study. A total of 10% chloral hydrate (0.4 mL/100 g) was injected intraperitoneally. The infrarenal segment of the abdominal aorta was exposed after complete anesthesia in mice, and the abdominal aorta and inferior vena cava were carefully separated. Instill 20 μL elastase (Sigma-Aldrich; 10.3 mg protein/mL, 5.9 U/mg protein) was around the exposed aorta adventitia. After incubating for 20 min, the abdominal cavity was flushed with normal saline three times, and the abdominal incision was sutured and disinfected. Following 28 days, mice were sacrificed. The abdominal aorta (vessel wall) was anatomized and divided into AAA and NAA groups and stored at −80°C until use.

### Peripheral Blood Samples With AAA Patients

A total of 55 peripheral venous blood samples (AAA vs. NAA = 40:15) were collected from patients in the First Affiliated Hospital of Zhengzhou University. Baseline clinical data and patient characteristics are shown in Supplementary Table S1. For all the study participants, venous peripheral blood samples were collected on admission, and the plasma supernatant was stored at – 80°C after centrifugation. This project was approved by the Ethics Committee Board of The First Affiliated Hospital of Zhengzhou University. All patients signed informed consent documents.

### Quantitative Real-Time PCR

Total RNA was isolated from human serum and mice abdominal aorta vessel wall tissues using RNAiso Plus (Takara, Dalian, China) according to the manufacturer's protocol. The RNA quality was evaluated using a NanoDrop One C (Thermo Fisher Scientific, Waltham, USA) ultramicro UV spectrophotometer, and the RNA integrity was assessed using agarose gel electrophoresis. Reverse transcription was performed using the PrimeScript RT reagent Kit (Takara, Dalian, China) with genomic DNA (gDNA) Eraser.

After removing the gDNA at 42°C for 2 min, the tissue RNA was reverse-transcribed into cDNA under the following conditions: 37°C for 15 min and 85°C for 5 s. Serum RNA was reverse-transcribed into cDNA using a RevertAid H Minus First-Strand cDNA Synthesis Kit (Thermo Fisher Scientific, Waltham, USA) under the following conditions: 25°C for 5 min, 42°C for 60 min, and 70°C for 5 min. The product was immediately stored at −80°C until use.

The qRT-PCR was performed on a QuantStudio 5 Real-Time PCR System (Applied Biosystems, Foster City, USA) using a Hieff qPCR SYBR Green Master Mix (Yeasen, Shanghai, China). qRT-PCR assays were performed in triplicate with the following conditions: (1) 95°C for 5 min and (2) 40 cycles of 95°C for 10 s and 60°C for 30 s. The relative expression of mRNA was calculated using the ΔCT (Ct mRNA-Ct GAPDH) method. The relative quantification values for mRNA were calculated by the 2^−Δ*ΔCt*^ method. The qRT-PCR primer sequences were provided in Supplementary Table S2. GAPDH was used as an endogenous control for normalization.

### Gene Set Enrichment Analysis and Immune Infiltration Evaluation

Before GSEA, we used the “limma” package (23) to calculate the value of log2 (fold change) between PVAT around AAA and NAA. GSEA was used to decipher the underlying biological mechanisms of the adipose tissues around AAA by KEGG and GO terms (Molecular Signatures Database, version: c2.cp.kegg.v7.4.symbols.gmt and c5.go.v7.4.symbols.gmt). To calculate immune cell infiltration abundance, we uploaded the gene expression matrix data to CIBERSORT. After that, we drawn a correlation heatmap by “corrplot” package to display the correlation of immune cells. Furthermore, “ggplot2” package was used to draw boxplots to visualize the differences in PVAT immune cell infiltration around AAA and NAA. A two-dimensional principal component analysis (PCA) clustering map was drawn based on the PCA clustering analysis on immune cell infiltration data (which was calculated by “factoextra” package).

## Results

### Identification of AAA-Related Modules by WGCNA

**Figure 2:**
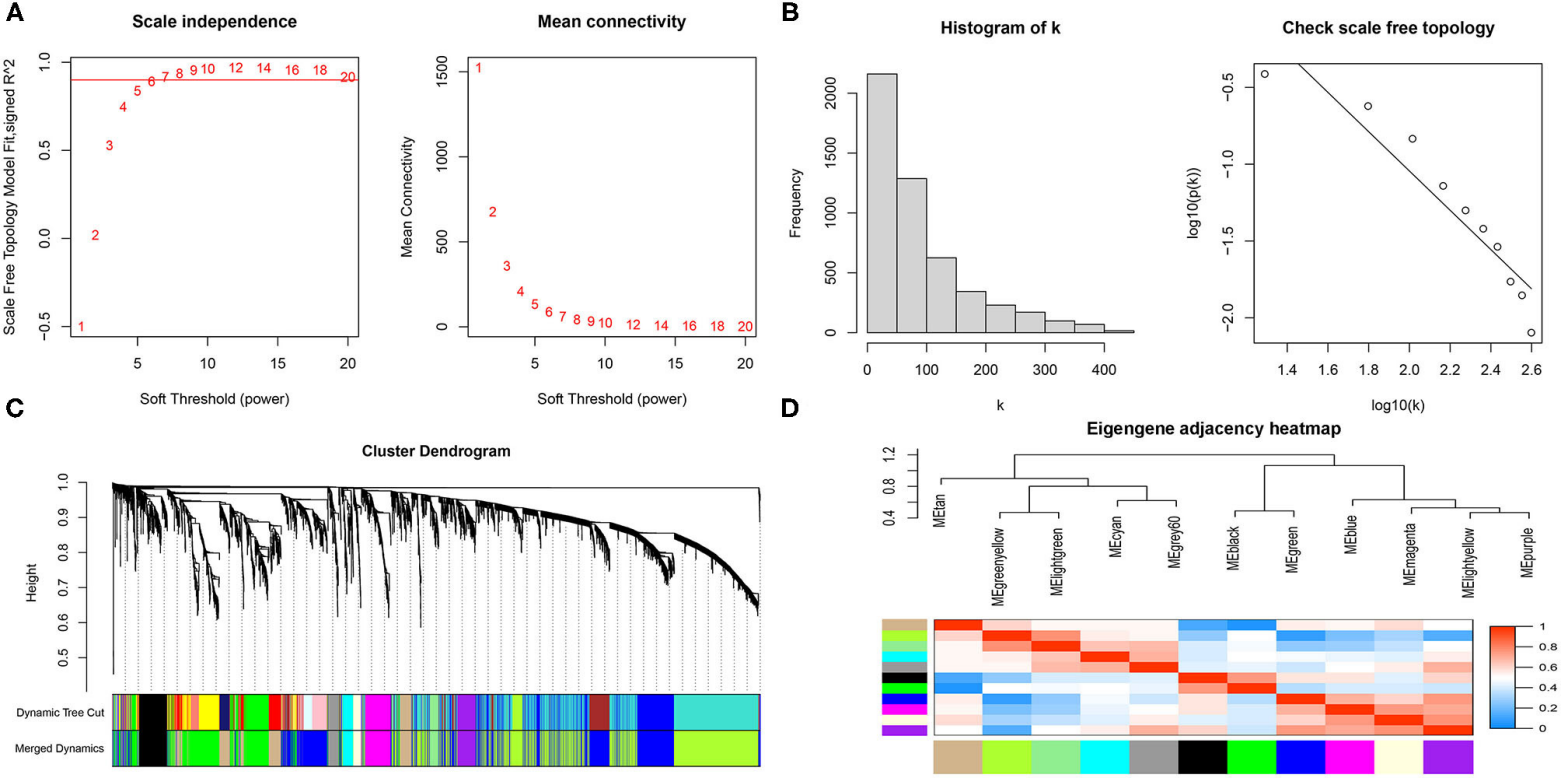
Scale-free networks were constructed and genes were clustered by WGCNA. **(A)** Scale-free network analysis under different soft-thresholding powers. The left panel shows that scale-free topological indices at different soft-thresholding powers. The right panel shows the correlation analysis between the soft-thresholding powers and average connectivity of the network. **(B)** Histogram of connectivity distribution and checking the scale free topology when β = 6. **(C)** Gene clustering diagram based on hierarchical clustering under optimal soft-thresholding power. **(D)** Heatmap of the eigengene adjacency.

**Figure 3:**
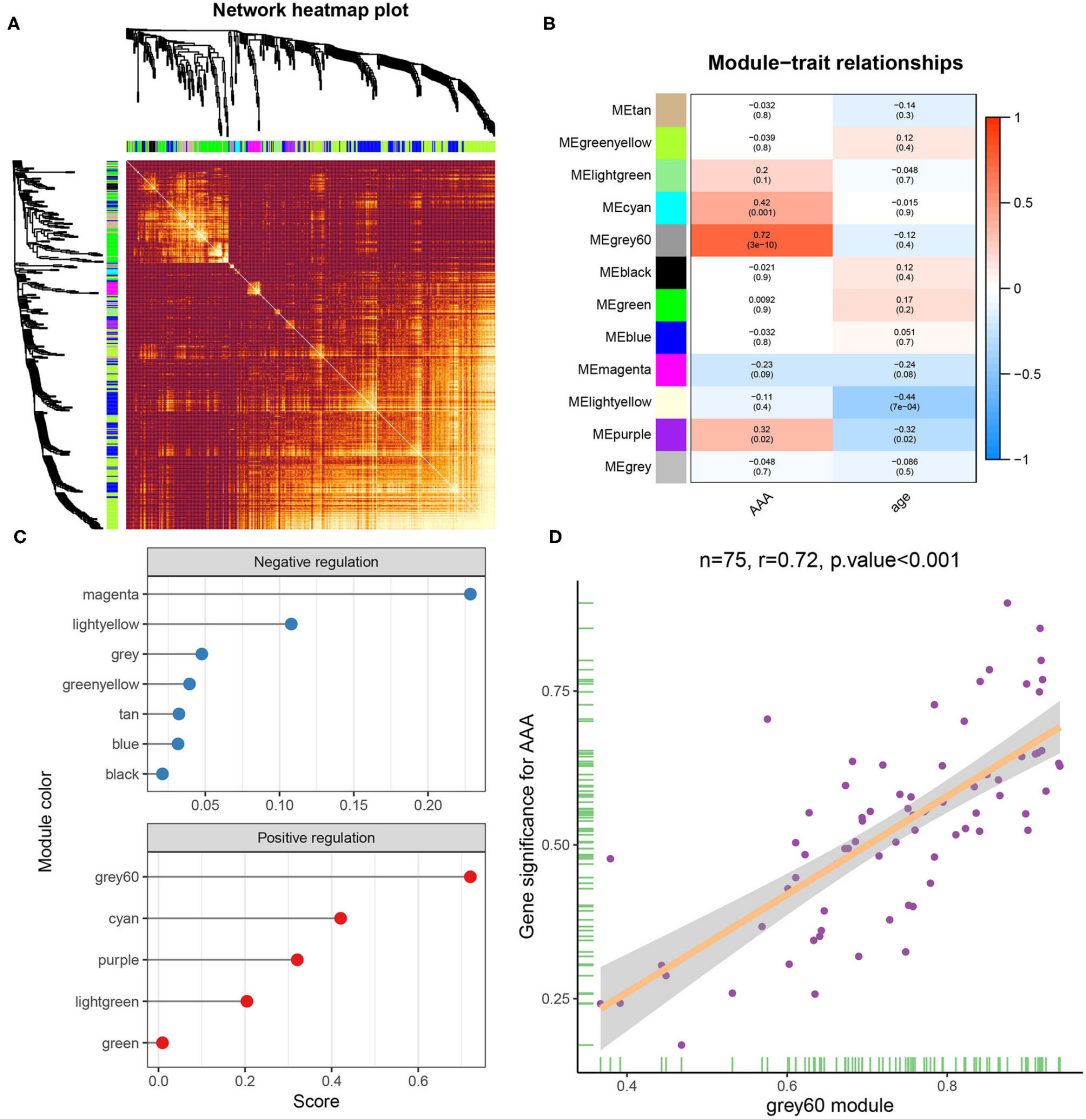
Gene module analysis based on WGCNA. **(A)** Heatmap of TOM of genes which selected by WGCNA. **(B)** Heatmap between gene modules and clinical characteristics. **(C)** Correlation score of modules with adipose tissue around AAA. **(D)** Correlation analysis between genes in grey60 module and adipose tissue around AAA.

The gene expression matrix of GSE119717 was obtained (14,016 genes) after data preprocessing. WGCNA analysis was carried out on top 5,000 genes which were selected based on the MAD. In detail, the soft-thresholding power in WGCNA was selected based on scale-free R^2^, and we chose six as the optimal soft threshold by the “pickSoftThreshold” function (Figures 2A,B). Subsequently, the coexpression modules in the network were identified by the “cutreeDynamic” function, and eleven modules were identified based on dynamic tree clipping and average hierarchical clustering (Figures 2C,D). The number of genes in every module is shown in Supplementary Table S4. Ultimately, the grey60 module was highly related to adipose tissues around AAA (Figures 3B,C); this module was selected as a key module for further analysis. The heatmap depicted TOM among 400 genes (which was randomly selected from total genes) in WGCNA (Figure 3A), and the scatterplot displayed correlation between PVAT and 75 genes in the grey60 module (Figure 3D).

### GO Annotation and KEGG Enrichment Analysis

**Figure 4:**
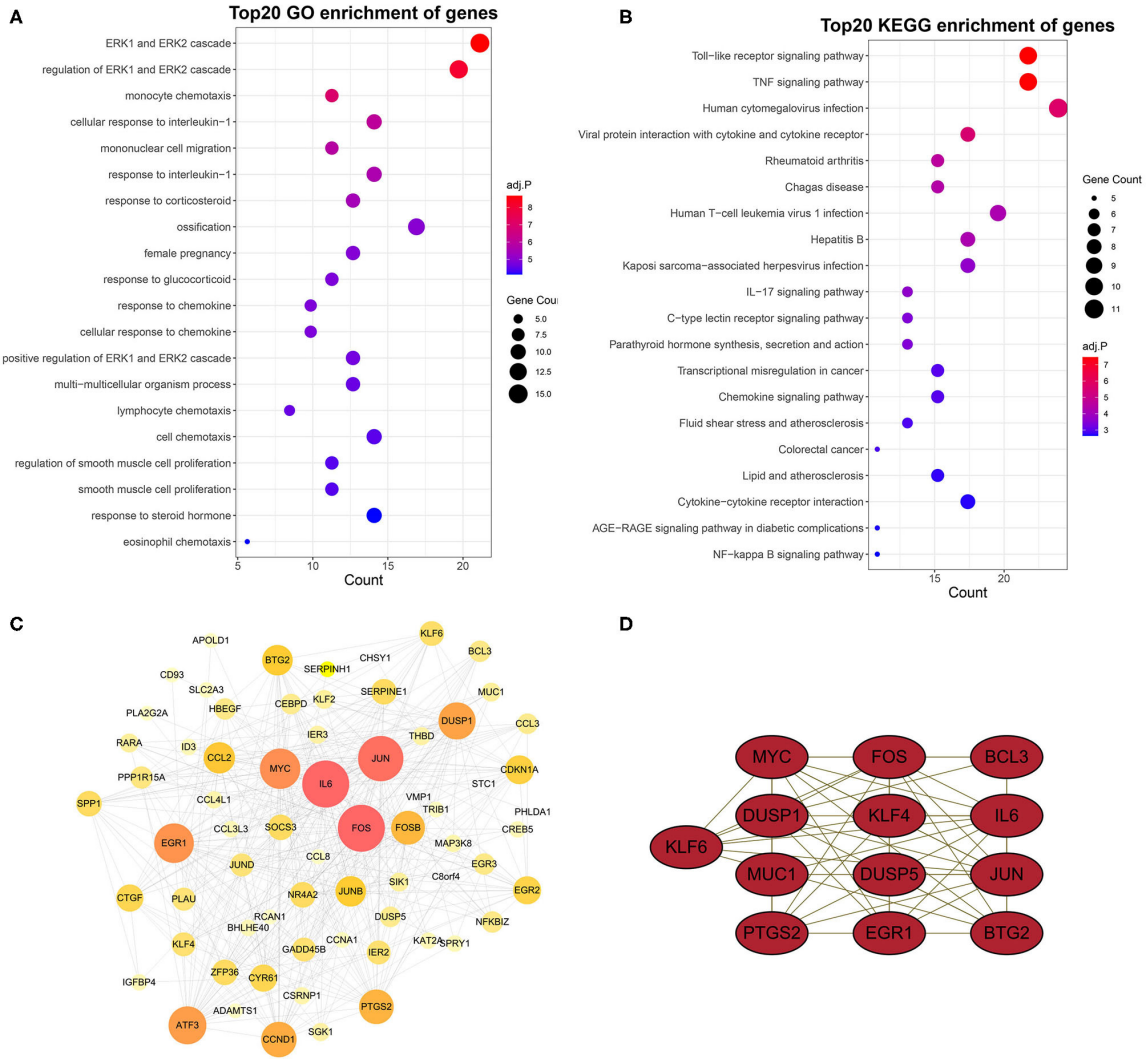
GO and KEGG enrichment analysis and PPI network and hub genes. GO **(A)** and KEGG **(B)** enrichment analysis of yellow module genes. **(C)** PPI network analysis of yellow module genes. Edge indicates that there is an interaction between two proteins. The greater the node indicates that the protein is more important in the network. **(D)** Most closely connected network cluster in the PPI network was identified using the MECOD plugin (score = 8.667, nodes = 13, edges = 52).

To obtain a better understanding of the functional features of 75 genes in the grey60 module, GO and KEGG enrichment analyses were performed by “enrichGO” and “enrichKEGG” function. Terms with *p* < 0.05 (GO and KEGG) were visualized with R language as a bubble plot. As shown in the results, terms such as *ERK1* and *ERK2* cascade, mononuclear cell migration, cellular response to interleukin-1, and monocyte chemotaxis were enriched in GO biological processes (Figure 4A). Furthermore, toll-like receptor signaling pathway and lipid and atherosclerosis were also significantly enriched (Figure 4B).

### PPI Network Construction

To identify the hub genes of these genes related to AAA in the grey60 module, we constructed a PPI network by STRING database and Cytoscape software (Figure 4C).

Subsequently, key modules in the PPI network were identified by MCODE plugin. The key module (score = 8.667, nodes = 13, edges = 52) consisted of 13 target genes, including *KLF6, MYC, FOS, BCL3, DUSP1, KLF4, IL6, MUC1, DUSP5, JUN, PTGS2, EGR1*, and *BTG2* (Figure 4D).

### Screening and Verification of Diagnostic Markers

**Figure 5:**
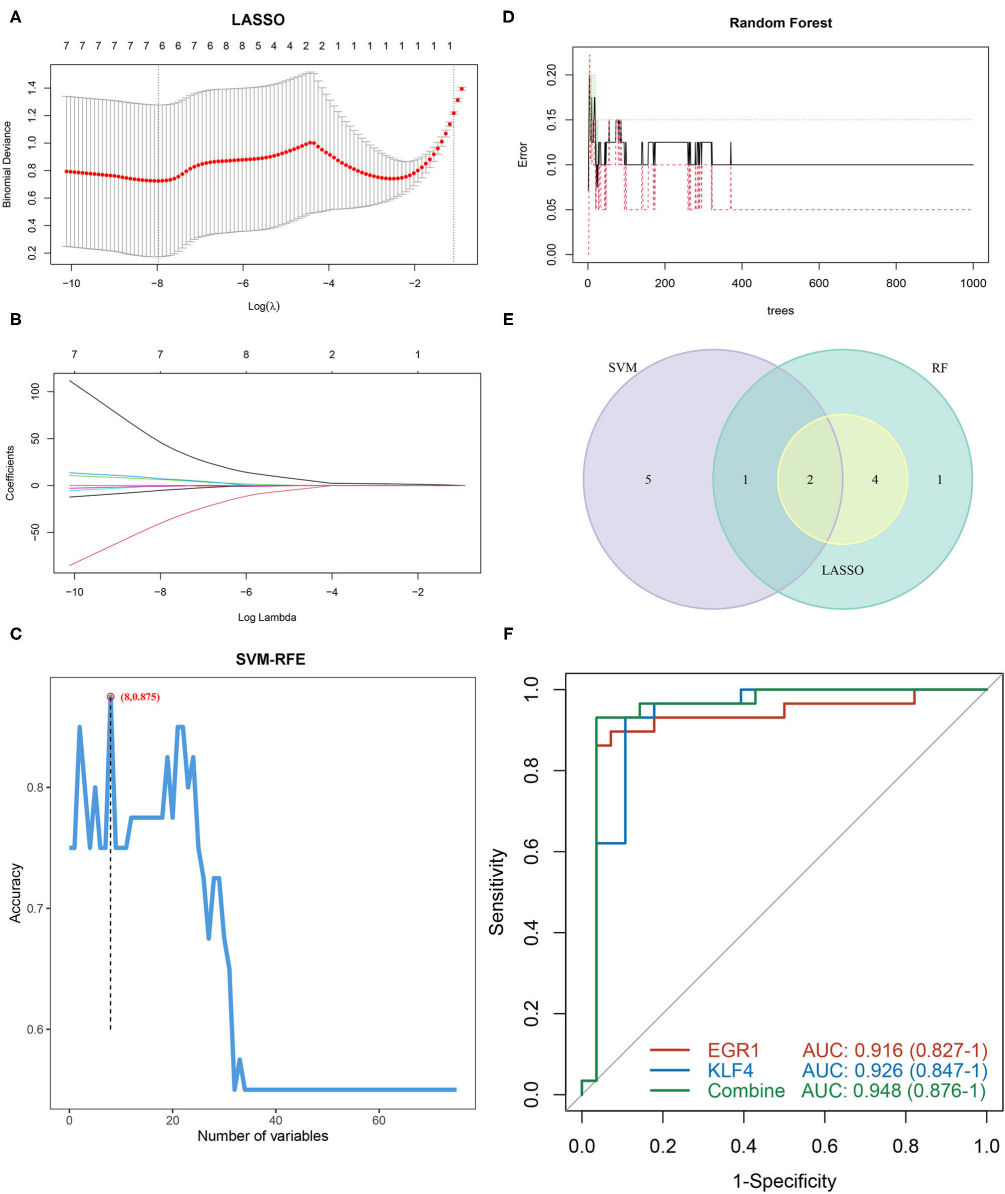
Screening and verification of diagnostic markers based on machine learning. **(A,B)** LASSO logistic regression algorithm to screen diagnostic markers. **(C)** SVM-RFE algorithm to screen diagnostic markers. **(D)** RF algorithm to screen diagnostic markers. **(E)** Venn diagram shows the intersection of diagnostic markers obtained by the three algorithms. **(F)** The ROC curve of the diagnostic efficacy verification after fitting two diagnostic markers to one variable.

To identify diagnostic markers for AAA, a variety of machine learning methods was utilized. First, we used the LASSO regression algorithm to identify six genes from the grey60 module as diagnostic markers for PVAT around AAA, including *EGR1, DUSP1, CYR61, FOSB, KLF4*, and *CREB5* (Figures 5A,B); eight genes were obtained from the grey60 module by the SVM-RFE algorithm as diagnostic markers, including *APOLD1, PLA2G2A, NR4A2, EGR1, KLF4, CCL8, CCL3L3*, and *FOS* (Figure 5C); we also used the RF algorithm to identity eight genes from the grey60 module as diagnostic markers, including *FOS, EGR1, DUSP1, CYR61, FOSB, JUN, KLF4*, and *CREB5* (Figure 5D). Subsequently, we overlapped the three sets of diagnostic genes and finally obtained two diagnostic markers (*EGF1* and *KLF4*) (Figure 5E). To further examine the diagnostic efficacy of *EGF1* and *KLF4*, ROC curve analysis was used and the AUC values were 0.916, 0.926, and 0.948, respectively, for *EGF1, KLF4*, and two-gene combined, respectively (Figure 5F) and the C-indexes all >0.9 (Supplementary Figure S1A).

### Validation of Biomarkers in Mice and Inhouse Cohorts

**Figure 6:**
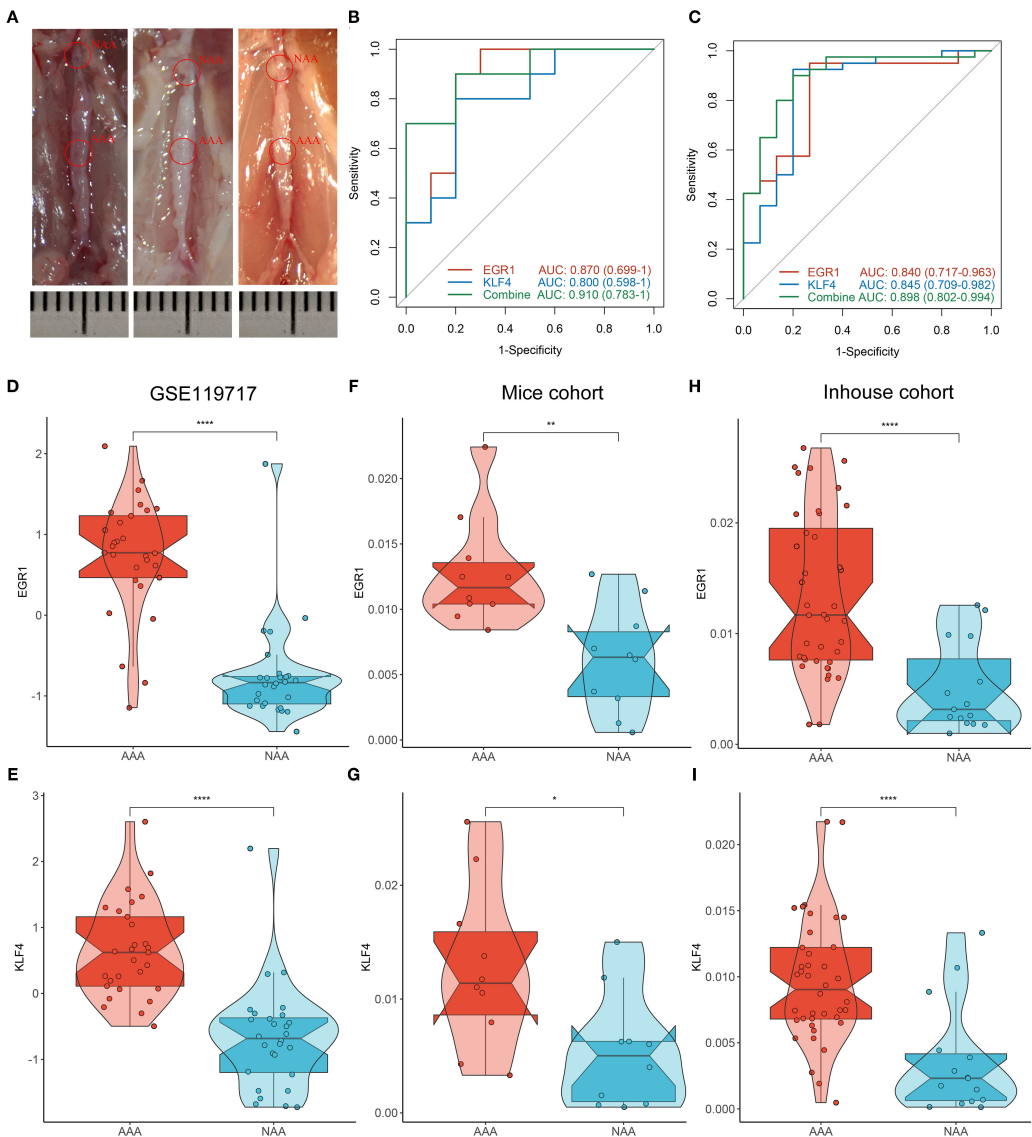
Validation of biomarkers in mice and inhouse cohorts. **(A)** Construct the AAA mice model. **(B,C)** The ROC curve of the diagnostic markers in the mice **(B)** and inhouse **(C)** cohorts. **(D–I)** Differential analysis of gene expression of *EGR1* and *KLF4* in the three cohorts. ^*^p < 0.05; ^**^p < 0.01; ^***^p < 0.001; ^****^p < 0.0001.

As illustrated in Figure 6A, we successfully constructed the AAA mice model. Subsequently, the evaluation of diagnostic efficacy of biomarkers was performed by ROC curve and C-index. The AUCs of *EGF1, KLF4*, and two-gene combined respectively were 0.870, 0.800, and 0.910 in the mice cohort (Figure 6B) and 0.840, 0.845, and 0.898 in the inhouse cohort (Figure 6C). The C-indexes of these diagnosis makers in the two cohorts were also larger than 0.8 (Supplementary Figures S1B,C), which *EGF1* and *KLF4* have high diagnostic and stability. By further analysis, we found significant differences in the expression levels of *EGR1* and *KLF4* among the GSE119717, mice, and inhouse cohorts (Figures 6D–I).

### Gene Set Enrichment Analysis

**Figure 7:**
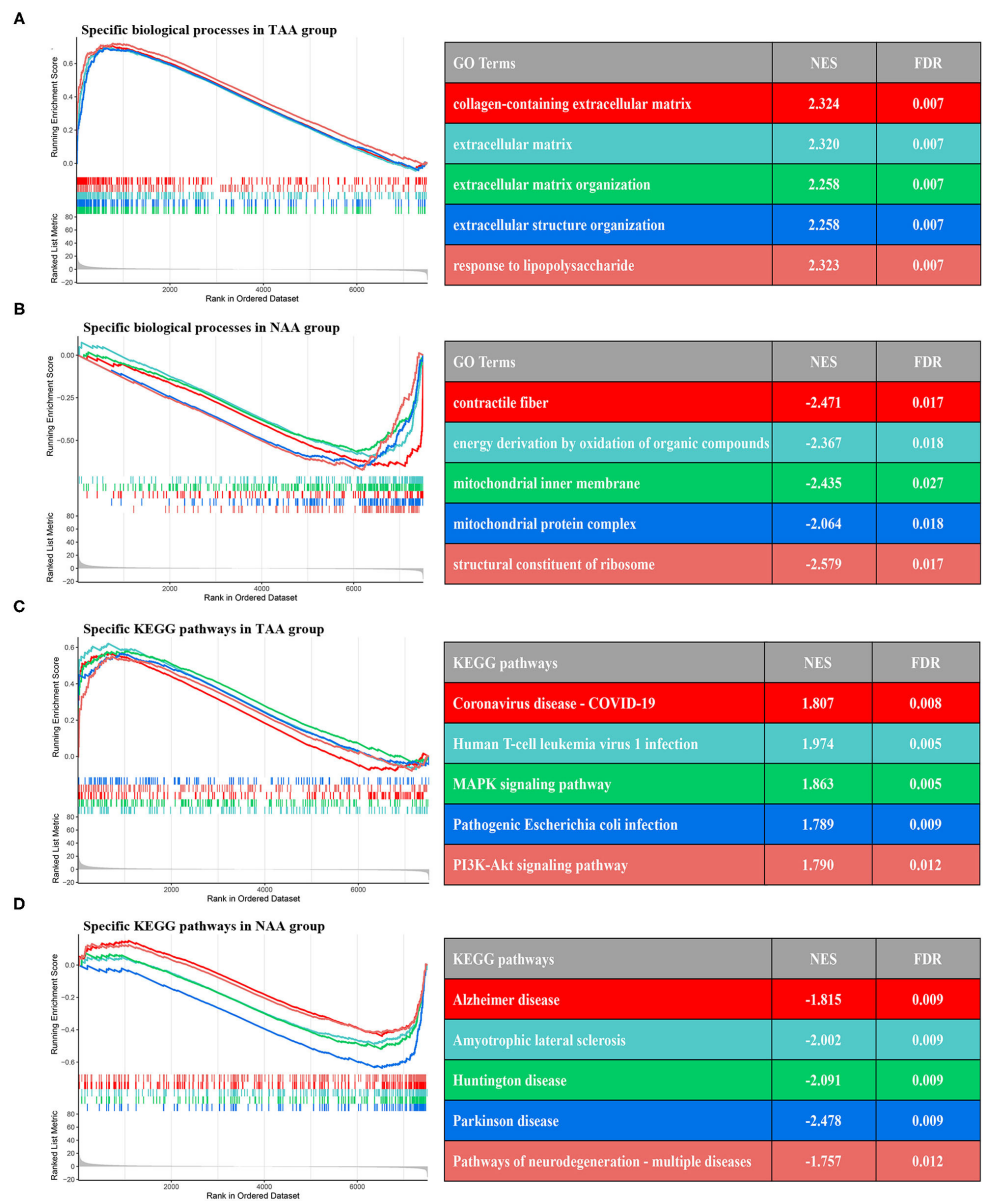
GSEA enrichment analysis between two subtypes. **(A,B)** The top five GO terms of differential genes in the two groups. **(C,D)** The top five KEGG pathways of differential genes in the two groups.

To characterize the relevant differences in gene expression between PVAT around AAA and NAA, we performed GSEA analysis based on the rank log2 (fold change) of all gene list. The results displayed that the significant GO terms mainly focus on extracellular matrix and fiber constitute (Figures 7A,B), such as “collagen-containing extracellular matrix” (normalized enrichment score (NES) = 2.324, FDR = 0.007), “extracellular matrix” (NES = 2.320, FDR = 0.007), contractile fiber (NES = −2.471, FDR =0.017), and structural constituent of ribosome (NES = −2.579, FDR = 0.017). Furthermore, the significant KEGG pathway (Figures 7C,D) mainly focus on “*MAPK* signaling pathway” (NES = 1.863, FDR = 0.005), *PI3K*-*Akt* signaling pathway (NES = 1.790, FDR = 0.012), and amyotrophic lateral sclerosis (NES = −2.002, FDR = 0.009).

### Immune Infiltration Analysis

**Figure 8:**
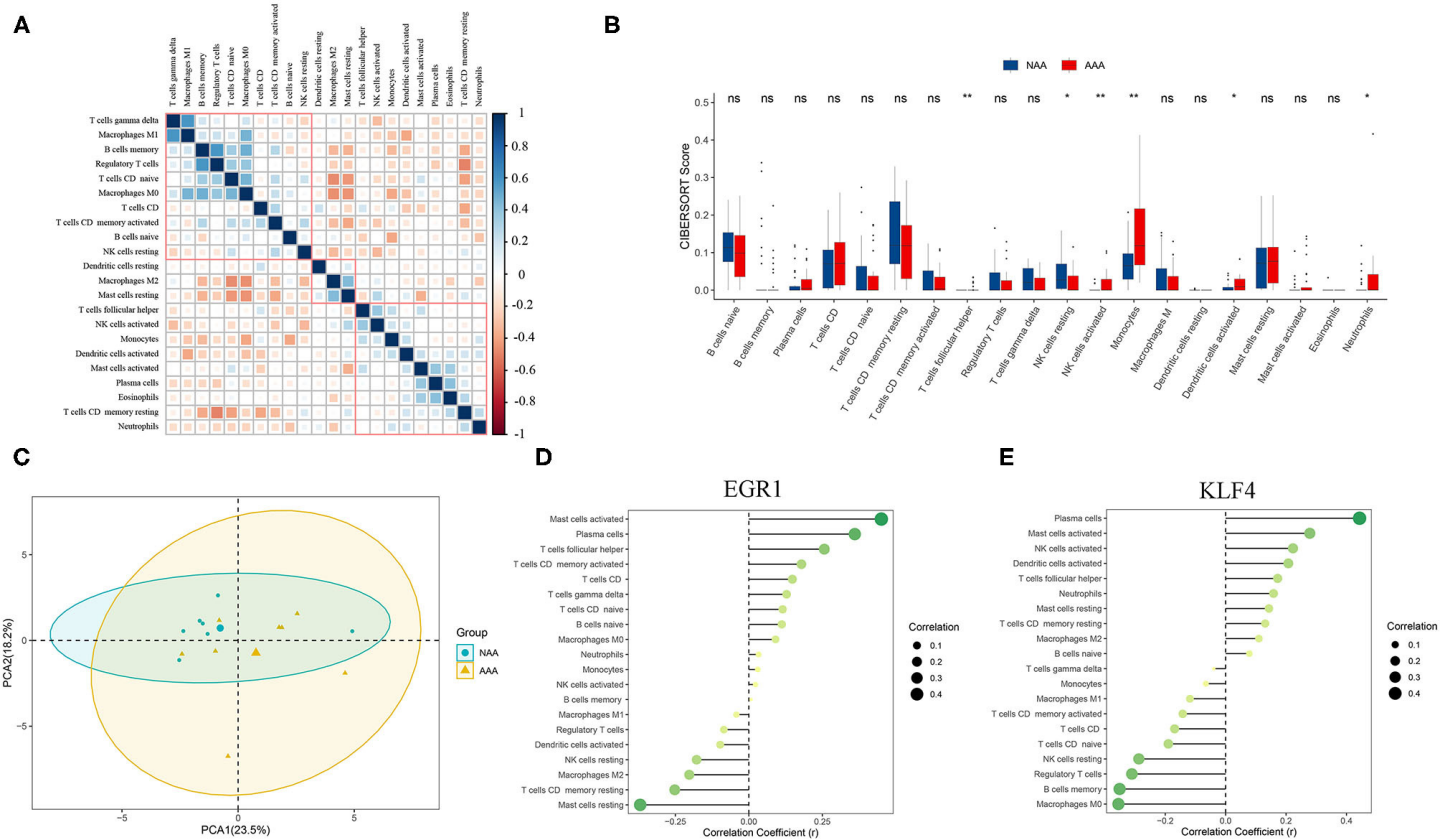
Analysis of immune infiltration between the two groups and correlation analysis between *EGR1, KLF4*, and immune cells. **(A)** Correlation heatmap of 22 types of immune cells. The size of the colored squares represents the strength of the correlation. Blue represents a positive correlation and red represents a negative correlation. The darker the color, the stronger the correlation. **(B)** Boxplot of the proportion of 22 types of immune cells (Wilcoxon test). **(C)** PCA cluster plot of immune cell infiltration between AAA samples and control samples. **(D,E)** Correlation between *EGR1, KLF4*, and infiltrating immune cells. ns, not significant *p* > 0.05; ^*^p < 0.05; ^**^p < 0.01.

The immune cells' score was calculated by CIBERSORT in two groups. Correlation heatmap (Figure 8A) of the 22 types of immune cells displayed that T cells gamma delta and macrophages M1, B cells memory, and regulatory T cells had a significant positive correlation. In contrast, T cells CD memory resting and regulatory T cells, T cells CD native, and macrophages M2 had a significant negative correlation. PCA revealed that there was a significant difference in immune cell infiltration between PVAT around AAA and NAA samples (Figure 8C). The boxplot of the immune cell infiltration difference displayed that, compared with NAA group, T cells follicular helper, NK cells activated, monocytes, dendritic cells activated, and neutrophils infiltrated were more abundant, whereas NK cells resting infiltrated less (Wilcoxon test, Figure 8B).

### Correlation Analysis Among EGR1, KLF4, and Immune Infiltration

Correlation analysis revealed that *EGR1* was positively correlated with mast cells activated (*r* = 0.451, *p* = 0.012) and plasma cells (*r* = 0.360, *p* = 0.02) and negatively correlated with T cells CD memory resting (*r* = −0.252, *p* = 0.007) (Figure 8D). *KLF4* was positively correlated with mast cells activated (*r* = 0.279, *p* = 0.028) and negatively correlated with macrophages M0 (*r* = −0.355, *p* = 0.003) (Figure 8E).

## Discussion

Due to the high frequency and lethality, considerable research has been invested to reveal the complex molecular mechanism of AAA, but the results were not satisfactory. Nevertheless, a neotype of adipose tissue that surrounds blood vessels (including abdominal aorta), known as PVAT, has been demonstrated by mounting evidence as an active component of the vascular wall regulating vascular homeostasis and influencing the pathogenesis of atherosclerosis (14, 34). Over the past decades, PVAT was not solely considered as a protective cushion for the abdominal aorta, but rather as a new endocrine and paracrine organ and secretes inflammatory cytokines to promote vascular wall remodeling and cause the formation of AAA (16). In addition, immune cell infiltration has been considered to serve as an important part in PVAT and aneurysm wall (17, 35, 36). Therefore, identifying the diagnostic markers and calculating immune cell infiltration abundance of PVAT are significant for understanding the cellular and molecular mechanisms of AAA and retarding the formation and rupture of AAA. On the other hand, as an advanced statistical approach, machine learning can use gene expression and clinical datasets to exploit accuracy models and identify diagnostic markers (37–39). With the development of bioinformatics technology, LASSO, RF, and SVM algorithms have the ability to accurately screen biomarkers, and CIBERSORT was recommended to analyze immune cell infiltration abundance of diseases.

In our study, to analyze the correlation between PVAT and AAA, we downloaded the PVAT clinical annotation and gene expression profiling dataset from the GEO database. We extracted a grey60 module (including 75 genes) significantly associated with PVAT through WGCNA. To further investigate the potential functions and mechanisms of the module, GO and KEGG enrichment analyses were practically applied. GO analysis showed that “*ERK1* and *ERK2* cascade,” “monocyte chemotaxis,” “regulation of smooth muscle cell proliferation,” “cellular response to interleukin-1,” and “eosinophil chemotaxis” were most significantly enriched functional modules. Meanwhile, KEGG analysis further revelated that “lipid and atherosclerosis.” “fluid shear stress and atherosclerosis,” “toll-like receptor signaling pathway,” and “IL-17 signaling pathway” were primary pathways. According to the above results, inflammation and immune-related pathways and functions are the most prominent enrichment in PVAT. Lu et al. (17) showed that infiltrating T cells were essentially always exist in PVAT and aneurysm lesions, and the proportion of CD4^+^/CD8^+^ in AAA was 2–4 times that of normal peripheral blood. Furthermore, regulatory immune cells and memory immune cells have been found to coexist in PVAT, and immune infiltration in PVAT was symmetrically positively correlated with the progression of AAA (18, 40, 41). The above researches have revealed that immune cells (especially T cell) played an important role in the formation and progression of AAA.

Afterward, we further identify diagnostic markers for PVAT and analyzed the association between them and immune cell infiltration. To prevent overfitting, which is a common problem in many machine learning algorithms (42), RF, LASSO, and SVM-RFE were selected. In this study, *EGR1* and *KLF4* were ultimately confirmed as the diagnostic markers of PVAT around AAA by the above three methods. As we know, oxidative stress in VSMCs can promote degeneration of aorta wall and ultimately lead to rupture (43). One study showed that the high expression of *EGR1* in AAA indicates that there was a correlation between *EGR1* and AAA. Further research showed that downregulation of *EGR1* in the lncRNA *Sox2ot* – miR-145 – *EGR1* pathway can inhibit VSMCs oxidative stress and inflammatory response in AAA, which decelerate the formation and progression of AAA (44). Wang et al. showed that *TRPV5* impeded the phenotypic switch of SVMCs by downregulating *KLF4* which in turn inhibits AAA formation and provided a new strategy for the treatment of AAA (45). In our study, *EGR1* and *KLF4* were highly expressed in PVAT, which supports the conclusion that PVAT was a risk factor for the formation and development of AAA. Therefore, evidences from previous studies and our research revealed that *EGR1* and *KLF4* were involved in the progression of AAA and may be potential targets for the treatment of AAA, but need further validation.

To reveal the differences in immune cell infiltration in PVAT, we used CIBERSORT to calculate the abundance of 22 immune cells in PVAT around AAA and NAA. Compared with NAA group, PVAT around AAA was more abundant in multiple immune cell infiltration (including T cells follicular helper, NK cells activated, monocytes, dendritic cells activated, and neutrophils infiltrated). This supports the hypothesis of vibrant immune cell proliferation in the AAA wall and PVAT (17, 18, 21, 46). Additionally, analysis of the correlation among *EGR1, KLF4*, and immune cells revealed that mast cells activated and plasma cells were significantly positively associated with *EGR1*, and macrophage M2 was significantly positively associated with *KLF4*. In contrast, macrophage M0 was negatively correlated with *KLF4*. Previous study has shown that TNF-alpha secreted by mast cells aggravated AAA and induced the formation of AAA in mice by secreting elastase (47, 48). The increase in plasma cells and macrophage contributes to the progression of the AAA (49, 50). Consequently, we hypothesize that the regulation of *EGR1* and *KLF4* promotes the proliferation of mast cells, plasma cells, and macrophage, which in turn promotes abdominal aorta dilation.

To our knowledge, our work was the first research to use machine learning to identity diagnostic markers of PVAT around AAA and delineate a comprehensive inflammation landscape. Our study has the following advantages: (1) Diagnostic markers were identified by multiple machine learning algorithms (including LASSO, RF, SVM, and MCODE). (2) We validated the diagnostic efficacy of biomarkers in the mice and inhouse cohorts, with both AUCs and C-indexes all >0.8. (3) Through immune correlation analysis, we found that the upregulation of *EGR1* and *KLF4* promoted the proliferation of multiple immune cells, thereby promoting the expansion of abdominal aorta. This provides a novel point for the prevention and treatment of AAA. However, this study has shortcomings as follows: (1) Due to the popularity of minimally invasive vascular techniques, the AAA tissue in humans is difficult to obtain. (2) Our works focused on mRNA levels and do not conduct in-depth exploration of noncoding RNA. We look forward to a large-scale international clinical cohort in the further to validate our results.

## Conclusion

In conclusion, we found that *EGR1* and *KLF4* are the diagnostic markers of PVAT around AAA by machine learning algorisms. Compared with PVAT around NAA, PVAT around AAA was abundant in immune cell infiltration. In addition, *EGR1* and *KLF4* were shown to have significant association with mast cells and plasma cells. Based on the results obtained, we think that the upregulation of *EGR1* and *KLF4* promotes the proliferation of immune cells, which promotes abdominal aorta dilation. Further studies of the complex interactions among *EGR1, KLF4*, and immune cells may provide new insights into the prevention and treatment of AAA.

## Data Availability Statement

The datasets presented in this study can be found in online repositories. The names of the repository/repositories and accession number(s) can be found in the article/Supplementary Material.

## Ethics Statement

The studies involving human participants were reviewed and approved by the Ethics Committee of The First Affiliated Hospital of Zhengzhou University. The patients/participants provided their written informed consent to participate in this study. The animal study was reviewed and approved by the Ethics Committee of The First Affiliated Hospital of Zhengzhou University.

## Author Contributions

CGG and ZL designed this work. CGG, ZQL, YY, ZBZ, KM, LFZ, and QD integrated and analyzed the data. CGG, LBW, LL, SZ, ZHH, and XWH wrote this manuscript. CGG and ZL edited and revised the manuscript. All authors approved this manuscript.

## Funding

This study was supported by the National Natural Science Foundation of China (81873527).

## Conflict of Interest

The authors declare that the research was conducted in the absence of any commercial or financial relationships that could be construed as a potential conflict of interest.

## Publisher's Note

All claims expressed in this article are solely those of the authors and do not necessarily represent those of their affiliated organizations, or those of the publisher, the editors and the reviewers. Any product that may be evaluated in this article, or claim that may be made by its manufacturer, is not guaranteed or endorsed by the publisher.
