# Supplementary figures and images for: EGR1 and KLF4 as Diagnostic Markers for Abdominal Aortic Aneurysm and Associated With Immune Infiltration
Source: Front Cardiovasc Med. 2022 Feb 9;9:781207. doi: 10.3389/fcvm.2022.781207 (PMC8863960; doi:10.3389/fcvm.2022.781207)

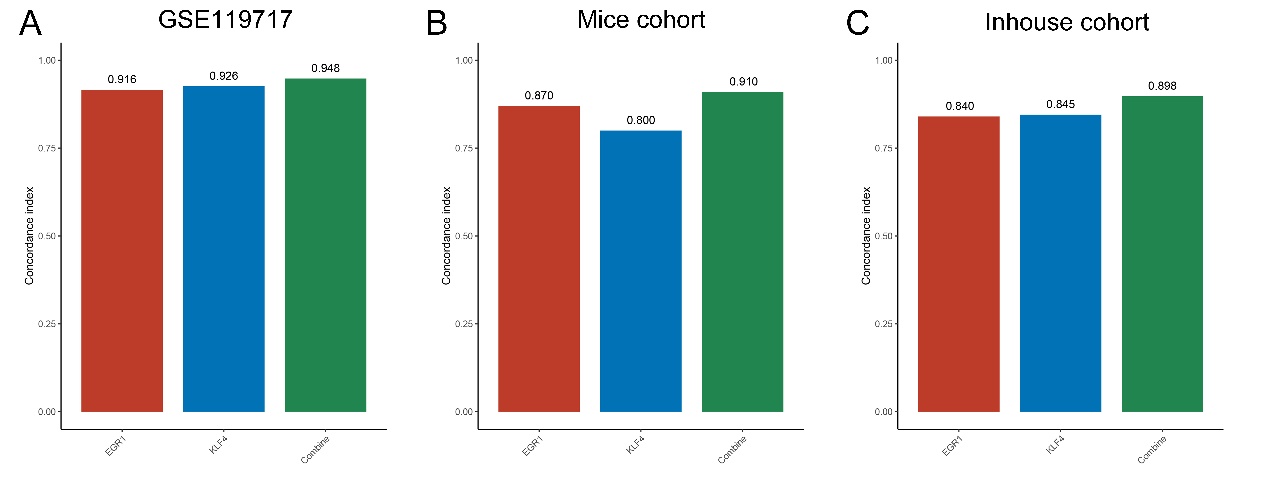


**Figure S1: The C-index of diagnostic markers in the GSE119717 (A), mice (B) and inhouse (C) cohorts.**

Supplement: Supplementary file 1 [file Table_1.DOCX]
